# Fragmented social networks promote complex behavioural contagions over infectious disease spread

**DOI:** 10.1098/rspb.2026.1424

**Published:** 2026-10-28

**Authors:** Nitara Wijayatilake, Shweta Bansal, Amy B. Pedersen, Matthew J. Silk

**Affiliations:** ^1^ Institute of Ecology and Evolution, School of Biological Sciences, The University of Edinburgh, Edinburgh, EH9 3FL, UK; ^2^ Department of Biology, Georgetown University, Washington D.C. 20057, USA

**Keywords:** social networks, complex contagion, animal behaviour

## Abstract

Group living and social interactions among animals provide key benefits, such as the exchange of beneficial social information and novel behaviours, but also often pose the risk of spreading costly infectious diseases, presenting a social trade-off. While both behaviours and infections spread across social networks, they typically have distinct mechanisms of transmission. Here, we model social behaviour spread as a complex contagion governed by a conformist learning rule, while pathogen transmission follows a simple contagion mechanism. Building on theoretical foundations, our study applies computational models to examine how (sub)group structure (modularity) influences the spread of these contagions across diverse animal social structures sourced from the Animal Social Network Repository. Our findings reveal that high modularity and (sub)group structure slow simple contagion spread, whereas complex contagions are less impeded by this fragmentation but with a relatively small effect size compared with theoretical predictions. Consequently, our results suggest that social networks divided into small groups or subgroups can help balance the competing pressures of exploiting beneficial social information and avoiding infectious disease in real-world networks.

## 1. Introduction

Animal social systems exhibit remarkable diversity, from largely solitary species to those forming stable groups and differentiated social bonds, with many intermediate forms of social system and intraspecific variation in interaction patterns [1–3]. Social interactions with conspecifics can offer significant advantages, including social transmission of information and behaviours [4,5]. Through social learning, individuals can acquire novel behaviours from their peers related to habitat selection, resource acquisition and anti-predator strategies, which can enhance their fitness [6–8].

However, sociality comes with costs, such as increasing the risk of pathogen and parasite transmission [9,10]. Individuals that live in groups face both the benefits and costs of social interactions. Dolphins, for example, exhibit extensive social learning behaviours related to foraging, hunting and communication. Young dolphins learn complex hunting techniques, such as ‘sponging’—a foraging behaviour involving sponges as tools—by observing their mothers [11]. However, their close social interactions also facilitate the spread of pathogens such as *Morbillivirus* [12]. The simultaneous yet opposing forces of pathogen transmission and behavioural adoption represent a social trade-off [13,14]. Effectively balancing access and use of beneficial social information with the avoidance of harmful infections is therefore likely to promote individual fitness [15–17] and potentially group persistence [13,18]. Despite this, we still understand little about how different animal social structures jointly influence social contagions and pathogen spread. Comparative resources, such as the Animal Social Network Repository (ASNR) [19,20], are uniquely suited to address cross-species questions by enabling different contagion processes to be examined across real-world social network structures and to evaluate their evolutionary implications in animal social systems.

The concept of a ‘contagion’ is employed to describe the spread of diseases or ideas from one individual to another. While both pathogens and behaviour are frequently modelled as contagions, the mechanisms governing their spread can differ substantially. The spread of a pathogen to a susceptible individual for a directly transmitted infection (spread through direct social contact) typically depends on increased exposure to infected individuals, with each interaction typically contributing independently to the likelihood of transmission [21]. In contrast, behavioural adoption which includes both the acquisition and use of social information often involves a more complex process, where the effect of each interaction is not independent but instead shaped by cumulative or reinforcing dynamics. Complex behaviours like tool use or mate choice may require multiple exposures or social reinforcement to spread effectively to new individuals [8,22,23]. For this reason, behavioural contagions are often modelled as complex contagions, where multiple exposures (i.e. repeated interactions with informed individuals) are required before transmission occurs, commonly formalized using threshold-based or nonlinear reinforcement models [24–27]. In contrast, directly transmitted infectious diseases are typically treated as simple contagions, where each contact with an infected individual carries an independent probability of spread [28,29]. This distinction accounts for nuances inherent to each transmission process [30–32]. For our study, we define simple contagions as processes where each exposure independently increases transmission probability (e.g. directly transmitted pathogens). We define complex contagions as processes where adoption probability depends nonlinearly on the proportion of adopting neighbours, requiring social reinforcement (e.g. behavioural adoption).

Animals may employ various learning strategies, such as copying the behaviour of familiar individuals or conforming to majority behaviours, to uptake social contagions within their groups [8]. One proposed rule is conformist learning, where the prevalence of the behaviour is what guides the adoption process, with an individual disproportionately more likely to imitate a behaviour when the majority of their neighbours have already adopted it [33,34]. In animal behaviour and sociology, this is colloquially termed ‘copy the majority’, and it is widely seen in the human and animal world [35,36]. Research in great tits, *Parus major*, found that social learning is preferred over asocial learning, with most individuals choosing to adopt the behaviour demonstrated by the majority of their social contacts [37]. In a different study, nine-spined sticklebacks exhibited a disproportionately strong preference for majority behaviour when choosing feeding sites, indicating a high level of conformist learning [38]. When social contagions spread through conformist learning, differences in social network structure could illuminate how groups balance the competing pressures of social information and infectious disease, an evolutionary trade-off highlighted in Romano *et al.* [16].

Many animal social networks are subdivided into discrete social communities (e.g. based on groups or subgroups) [39,40], and these group structures are therefore of particular ecological interest. Here, we refer to communities as densely connected subgroups with more connections within than between groups [41] and modularity as a measure of the strength of such subdivision in a network [42]. Within communities, the high density of connections can intensify the spread of contagions [43]. Community structure can influence both disease [44–46] and behavioural [47] contagions. More broadly, experimental evidence demonstrates that modular network structure can limit the spread of perturbations by containing their effects within subgroups [48]. Sah *et al.* [46] found that infection burden was lowest in social networks with high modularity and fragmentation, where subgroups acted as structural barriers under Susceptible-Infected-Recovered (SIR)-type infection spread. However, independently of community size, these effects only became pronounced at very high modularity levels. Interestingly, owing to social reinforcement, complex contagions governed by threshold dynamics (where adoption occurs once a critical proportion of social contacts have adopted) have been shown to spread most effectively at intermediate levels of modularity, which balance dense intra-community ties with sufficient inter-community connectivity [47]. Understanding how social structures mitigate selection pressures—minimizing disease transmission while enhancing the spread of beneficial behaviours—could offer valuable insights into how these ecological dynamics contribute to the evolution of group and subgroup structure in animal social systems.

Evans *et al.* [32] revealed that highly modular networks with small groups favoured the spread of a conformist over a simple contagion, suggesting that networks with numerous, small subgroups might regulate the trade-offs between behavioural and infection transmission within the same set of social interactions. However, the idea that modular network structures may help balance the trade-off between infection and behaviour spread has not been tested in real-world systems. This gap restricts our understanding of how natural social systems balance competing selection pressures on sociality. To address this, we draw on the unique breadth of data offered by the ASNR to compare the speed of spread for both simple and complex contagions within diverse real-world networks [19]. The ASNR enables a comparative framework that captures cross-species variation, allowing evaluation of whether theoretical predictions about contagion dynamics generalize across natural social systems [49]. We expect that community-structured networks with high modularity will slow the spread of both contagions, with this effect being most pronounced when the number of communities is highest (i.e. subgroups are smaller). Additionally, we expect complex behavioural contagions to spread through modular structures more effectively than simple infection contagions, resulting in their speed of spread being less impeded by community structure. Simulating contagion spread on real-world networks offers critical insight into how realistic social structures influence the interplay between pathogen and behavioural transmission, capturing dynamics that are difficult to observe in purely theoretical models. Therefore, by modelling contagions through empirical animal social networks, our study bridges the gap between theoretical and real-world structures, helping to quantify the potential for animal social group structure to influence the ecological consequences of social interactions.

## 2. Methods

All analyses were performed using R version 4.4.2 [50]. All code is available at our Zenodo repository [51].

### (a) Animal social network repository

Empirical networks were accessed from the ASNR version 3.0 [19,20], an open-source mixed species data repository of animal social behaviour networks spanning several taxonomic groups including insects, fish, birds, reptiles and mammals. We selected networks from the top three quartiles of the node count distribution in the ASNR (as≥ 10 nodes and as≥ 0.1 density) to ensure the networks were large enough to support contagion dynamics and subgroup formation, reducing our dataset from 1030 to 712. Next, we restricted our analysis to weighted networks, yielding a final dataset of 580 networks spanning 65 species and exhibiting a wide range of fragmentation (number of subgroups) and modularity values (figure 1). Supplementary figure 1 shows that the networks span a representative range of characteristics across ASNR. Simulations were performed on the largest connected component of each network, ensuring that contagions could feasibly reach all individuals. On average across networks, the largest connected component retained 92% of nodes.

**Figure 1.**
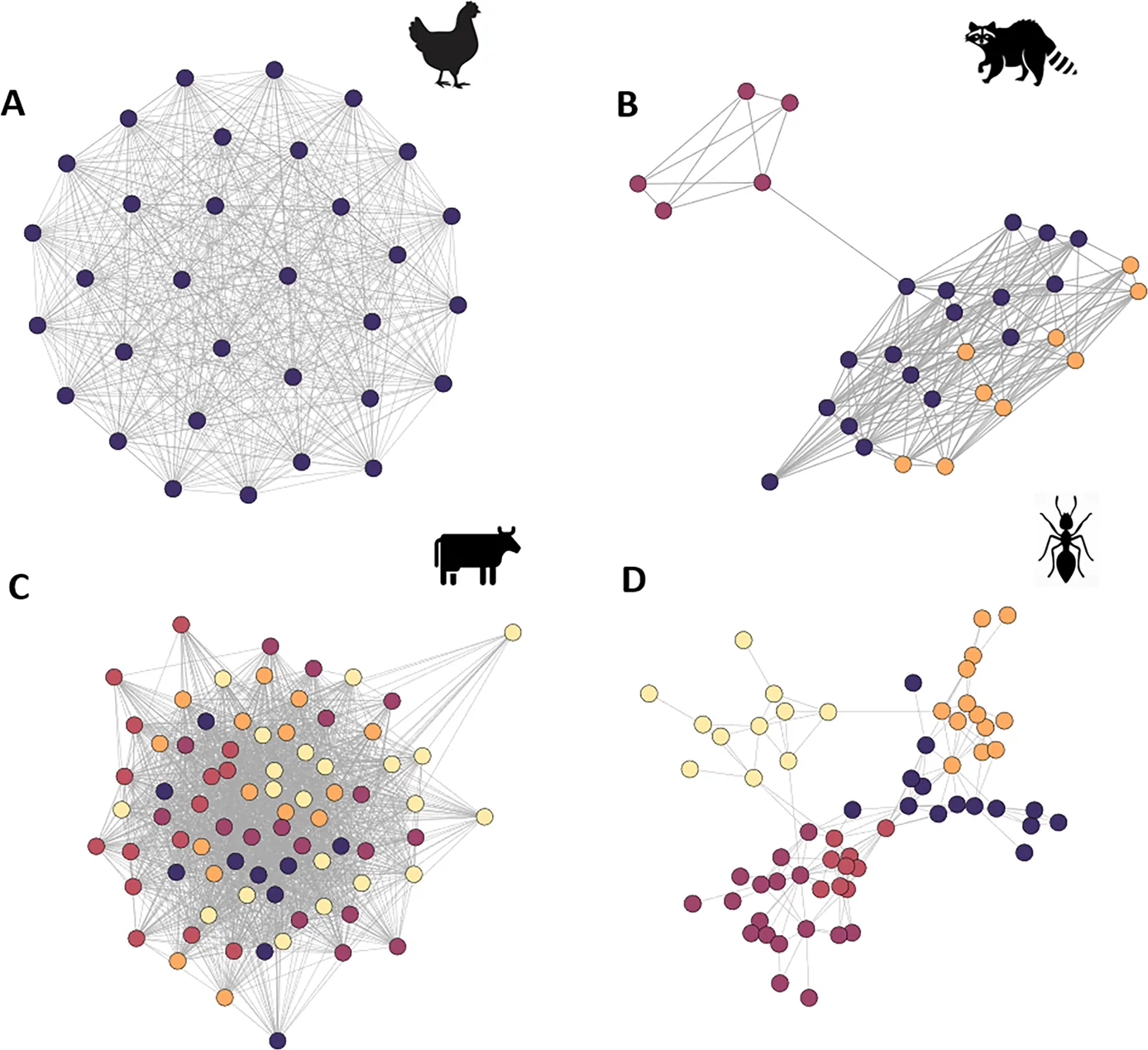
Example networks from the ASNR, demonstrating variations in modularity and community structure. Communities were detected using the Louvain community detection algorithm; colours denote communities and networks were plotted using igraph's default layout_nicely layout. (A) *Gallus gallus domesticus* (chicken): modularity = 0, single community. (B) *Procyon lotor* (raccoon): modularity = 0.4, three communities. (C) *Bos taurus* (cow): modularity = 0.1, five communities. (D) *Camponotus pennsylvanicus* (ant): modularity = 0.5, five communities.

### (b) Contagion spread

A susceptible-infected (SI) model was used to simulate the spread of both simple and complex contagions. The model focused solely on the transmission phase, to directly compare the transmission times of simple and complex conformist contagions. Baseline networks were generated using Erdős–Rényi random graphs, designed to match the size (number of nodes) and edge density of the largest connected component of each empirical network. Erdős–Rényi graphs were used as a structure-free baseline, following [32], to facilitate comparison with existing theoretical work and to calibrate transmission times between contagion types under identical size and density. This provides a consistent reference point, allowing differences observed on empirical networks to be attributed to network structure rather than differences in transmissibility. The simulation was seeded at a random node in the network and allowed to spread through the network with a given transmission probability of a node changing from susceptible (uninfected) to infected status. Since simulations used weighted networks, stronger associations increased the likelihood of contagion spread. We reran simulations with two and three initial seeds to assess sensitivity to seeding. Results were qualitatively consistent across seeding tests. We retain the single-seed results in the main text, as some networks are small and this setting better captures early-stage transmission dynamics (supplementary material).

For the simple contagion (pathogen) transmission, for each time step, the probability of infection for a node v is:

(2.1) $$1-(1-\beta {)}^{\left|N(v)\subseteq i\right|},$$

where β is the transmission rate and |N(v)⊆i| is the weighted sum of infected neighbours.

In our SI model, *R_0_* is defined as the basic reproduction number—that is, the average number of secondary infections produced by an infected individual in a fully susceptible network where all nodes are initially uninfected except the seed node as per [32] over a fixed time interval. We calculate the per-time-step transmission probability β from $R_{0}$ and the network's degree distribution as:

(2.2) $$\beta =\frac{R_{0}}{\frac{\left\langle k^{2}\rangle -\langle k\right\rangle }{\left\langle k\right\rangle }}\;,$$

where ⟨k⟩ is the average degree and $\left\langle k^{2}\right\rangle$ is the average squared degree. This adjusts for how the network's connectivity influences transmission. An $R_{0}$ of 1.5 was chosen based on findings from Evans *et al.* [26], which demonstrated that intermediate transmissibility values resulted in the greatest differences in transmission rates between behaviour and disease transmission. To ensure that findings were not driven by the choice of $R_{0}$, we reran simulations using a transmission probability calculated from $R_{0}$ of 3 and obtained qualitatively similar results (supplementary material).

We model behavioural transmission as a complex contagion using a conformist learning rule, where the likelihood an individual adopts a behaviour is determined by a sigmoidal function of the proportion of its neighbours that are informed, meaning that an individual only becomes more likely to adopt the behaviour contagion when a high proportion of its contacts are informed (and not just because it has more contacts overall). Figure S3 in the supplementary material illustrates the resulting nonlinear uptake dynamics across values of γ (explained below), which scales the overall rate of adoption without altering the sigmoidal response to social reinforcement.

In each time step, the probability of node v becoming informed is:

(2.3) $$\gamma \times \left(\frac{1-2\eta }{1+\exp\left(-\psi \times \frac{1}{\gamma }\times \left(\frac{\left|N(v)\subseteq i\right|}{\delta (v)}-0.5\right)\right)}+\eta \right),$$

where γ is the scaling parameter to modify the rate of behavioural contagion spread, |N(v)⊆i| is the weighted sum of informed neighbours, δ(v) is the node's degree, η is the base learning rate (to allow for a small amount of independent learning) and ψ is the steepness of the sigmoidal function of behavioural contagion uptake [32]. After exploring the parameter space, we set η at 0.001 to allow minimal independent learning, while a ψ of 10 effectively captured the steep dynamics of conformist learning.

To simulate transmission through social networks, we adapted the method from Evans *et al.* [32]. We measure the time for 75% of the network to become infected to capture the speed of contagion spread through the majority of the network. We also measure the time to 50% infection, but as this produced qualitatively consistent patterns (supplementary material), we report the 75% threshold as it better reflects spread across a larger proportion of the network. To determine an appropriate value for γ, the spreading parameter governing the rate of behavioural contagion, we first calibrated contagion dynamics within the baseline (random) networks. This calibration allowed us to select a γ value that ensured both pathogen and behavioural contagions spread at equivalent rates under identical network conditions. Doing so was important because, when later moving simulations into the empirical structures, we could isolate and examine the effects of community structure on the differences in dynamics between the two contagion types, rather than attributing differences solely to transmissibility.

To achieve this, we began by running 50 simulations of pathogen spread on the baseline networks, recording the time required for 75% of individuals in the network to become infected. This established a benchmark for simple contagion dynamics. Next, behavioural contagion spread was calibrated by testing a range of γ values, recording the time it took for 75% of individuals in the network to adopt the social contagion. We used the Bhattacharyya distance (a metric for quantifying the similarity between probability distributions) to evaluate the overlap between the spread time distributions of pathogen transmission and those of behavioural contagion for each γ value [52]. This approach enabled us to identify the optimal γ value that most closely aligned the extent of behavioural contagion spread with pathogen spread. Using the chosen optimal γ value, we then simulated the concurrent spread of infection and behaviour across each empirical network. Each simulation was repeated 50 times per network, seeding in a randomly selected node each time, and we recorded the time required for 75% of the network nodes to become either infected with disease or informed with a behavioural contagion. Figure 2 summarizes the steps involved in our simulation processes.

**Figure 2.**
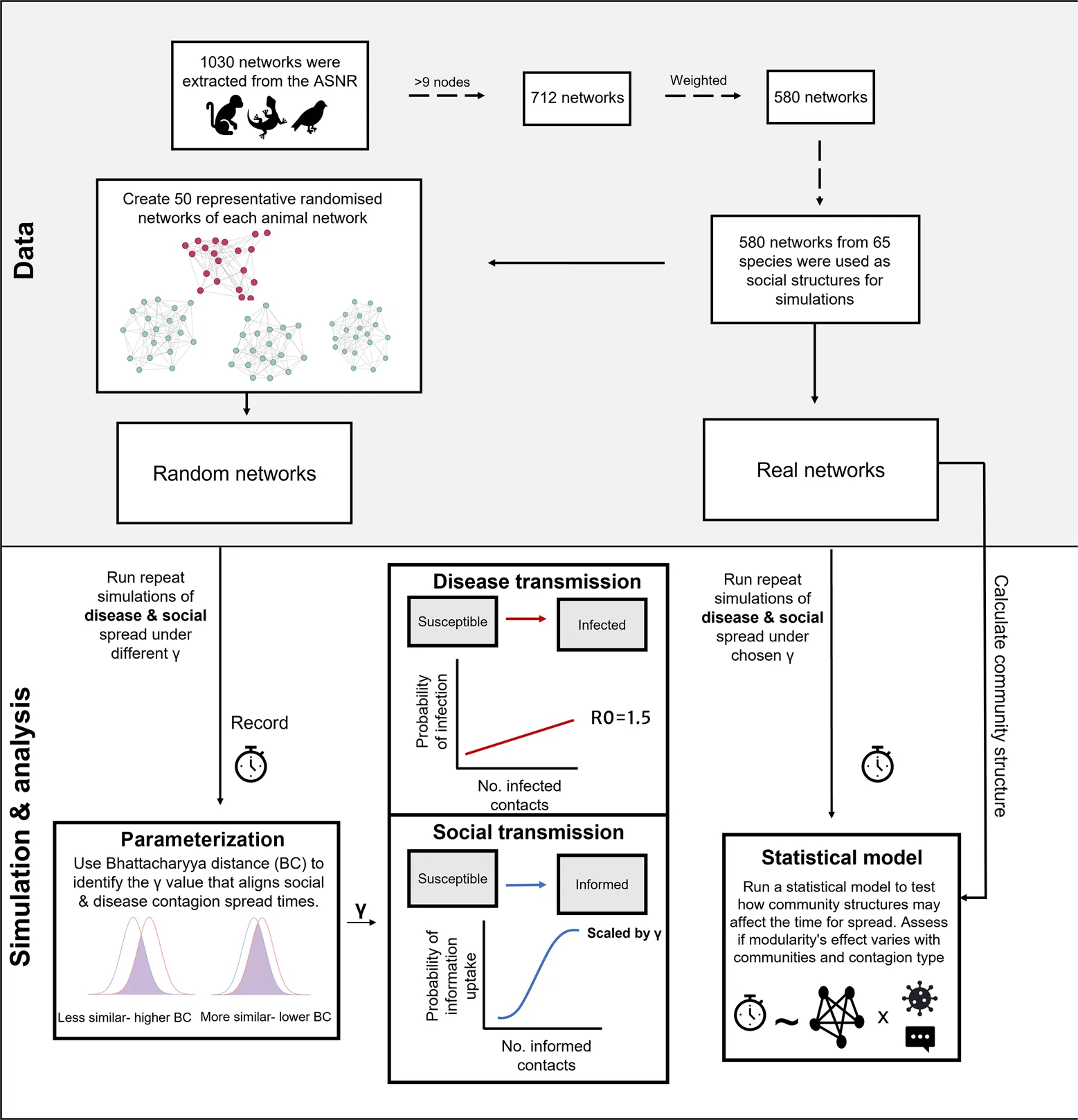
Overview of data extraction and simulation pipeline.

### (c) Network measure calculations

To investigate network structure as a predictor of relative contagion transmission differences, we calculated modularity, subgroup cohesion, network size (the number of nodes), edge density (proportion of possible edges present), variation in community size (coefficient of variation) and network fragmentation (the number of communities) of the empirical networks. Average module size and clustering coefficient were also calculated but excluded from analyses owing to high collinearity (Pearson's *r* >0.8) with other variables (supplementary material, figure S1). These measures were calculated using igraph [53]. Subgroup cohesion was measured as the proportion of total contacts that occur within subgroups [46]. For community detection, we employed a multi-level modularity optimization algorithm with cluster louvain [41] using default parameters. The number of communities served as an index of network fragmentation, reflecting both the number of subgroups and their relative sizes; as network size is controlled, a higher community count corresponds with smaller subgroup sizes. Modularity ( Q) was calculated following Newman's (2004) definition, which compares the density of edges within versus between communities [42], while $Q_{\text{rel}}$ was computed using the formulation of Sah *et al.* [46] to account for variations in network size and density (supplementary material).

### (d) Statistical model

We employed Bayesian regression models using the brms package in R to investigate how network structure influences the time taken for the contagion to reach 75% of the network for disease and behavioural contagions [54]. The response variable was the time required for contagion spread to reach 75% of the population, with fixed effects for contagion type (pathogen versus behavioural), modularity, number of communities, network size and edge density, as well as interactions among modularity, number of communities and contagion type. All numeric predictors were centred to improve interpretability. To account for the hierarchical structure of the data, we included random intercepts for networks nested within species. This approach allows the model to capture variability in spread times both between species and among networks within species. We allowed the model's residual standard deviation to vary across species and networks by modelling σ with random intercepts for networks nested within species; this accounts for potential heterogeneity in residual variance. We also ran a model with nested random effects for species within networks while assuming constant residual variance across groups. Model fit was assessed using Leave-One-Out Cross-Validation [55] and the model that allowed residual variance to vary across species and networks was selected (supplementary material). A skew-normal response distribution family was used to accommodate slight asymmetry in the tails of contagion spread times. Posterior distributions were estimated using the default No-U-Turn Sampler algorithm, implemented across four Markov chains. We conducted posterior predictive checks using density overlays to evaluate how well the model captured the observed data distribution (supplementary material, figure S4).

To further characterize community structure, we fitted an additional model including variation in community size and its interactions with number of communities and contagion type. We also assessed robustness by repeating analyses using different numbers of starting seeds, a 50% epidemic endpoint and transmissibility corresponding to $R_{0}=3$, and by replacing Q_rel_ with subgroup cohesion as an alternative measure of modular structure [46]. We also conducted two sensitivity analyses. First, we excluded *Camponotus fellah* networks, which contributed a disproportionate number of observations ( n=221), to assess their influence on the results. Second, because smaller and sparser networks showed greater variance in spread times, we repeated analyses using only networks with at least 20 nodes and density ≥0.15 (supplementary material). The main text presents results from all 580 networks to maximize ecological representation. Across all robustness checks, the direction of the main effects was unchanged, with behavioural contagions consistently spreading more rapidly in fragmented and modular networks (supplementary material).

## 3. Results

The selected networks from the ASNR represent a wide diversity of network structures (table 1). Approximately 45% of networks had Q_rel_ scores between 0.5 and 0.7 and between three and four communities. Figure 3 shows that our sample of real-world animal social networks span a broad range of variation in potential animal social structures.

**Figure 3.**
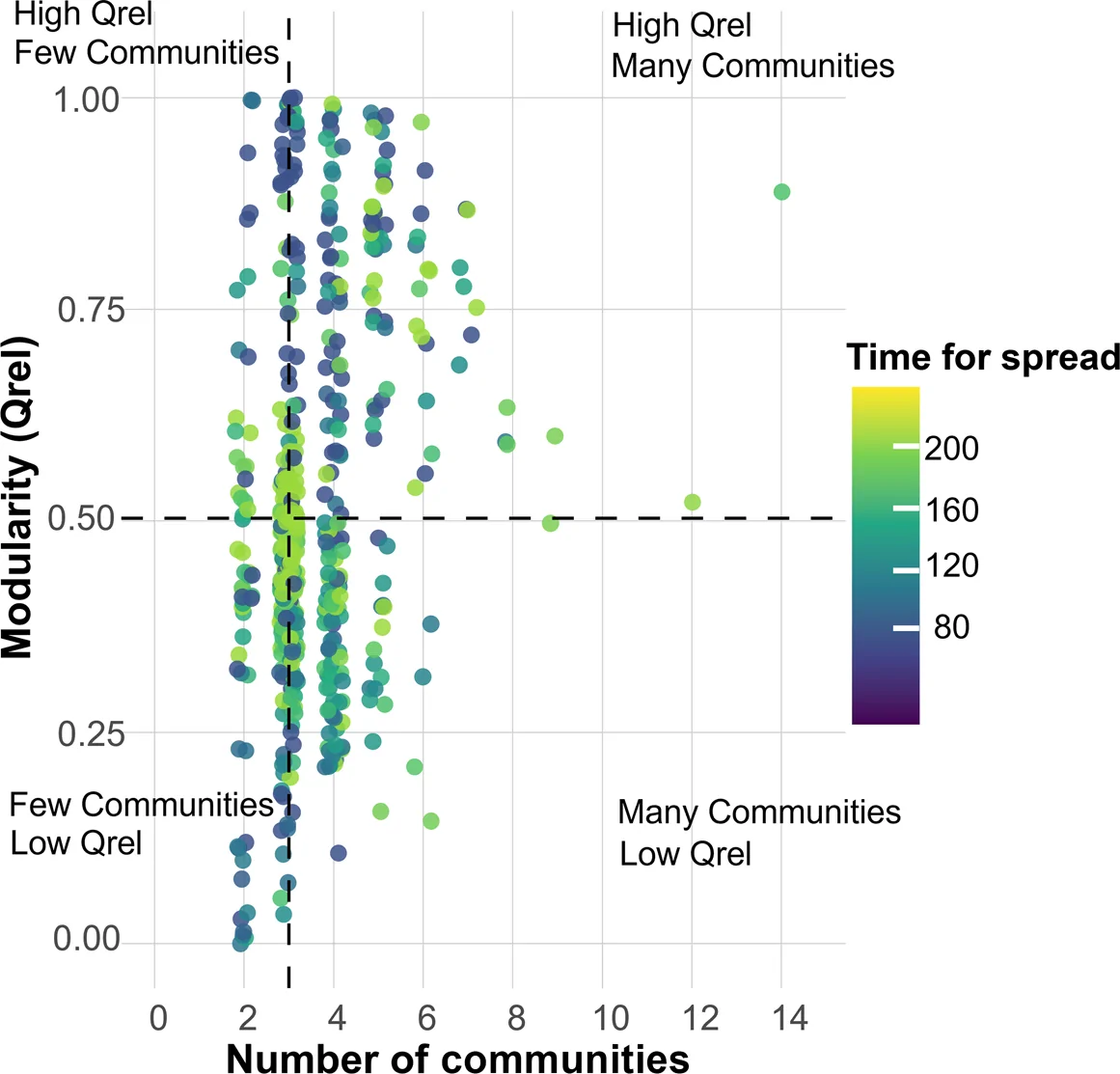
Relationship between modularity (Q_rel_) and number of communities across networks. Dashed lines indicate median values. Quadrant labels highlight combinations of (Q_rel_) and community number. Points are coloured by the time taken for contagion spread to reach 75% of the network. Networks with number of communities = 1 are excluded, as Q_rel_ is not meaningful for a single community.

**Table 1.** Summary of ASNR network characteristics selected for our model.

**metric**	**range**	**central tendency**
Q_rel_	0.00–1.00	mean = 0.51
number of communities	1–14	median = 3
edge density	0.10–1.00	mean = 0.52
network size	10–1007	median = 31

Our model results showed that networks with stronger community structures, indicated by higher modularity and more communities (higher fragmentation), took more time for 75% of the network to become infected by disease contagions. By contrast, behavioural contagions reached 75% of the network in less time as network fragmentation increased, demonstrating that complex contagions more effectively traverse community boundaries than disease contagions. The three-way interaction among contagion type, modularity and number of communities ( β=−16.87,(95%CI:−19.79,−13.99)) indicated that it is the combination of modularity and network fragmentation (number of communities) that affects contagion dynamics in animal social networks. When networks had few communities, the spread of both disease and behavioural contagions was unaffected by modularity. By contrast, in networks with many communities, behavioural contagions were better able to overcome the combined effects of high modularity and fragmentation, while disease contagions were increasingly delayed. Behavioural contagions even spread faster in more fragmented and modular networks (figure 4). These findings indicate that modularity strongly influences contagion dynamics only when fragmentation is high.

**Figure 4.**
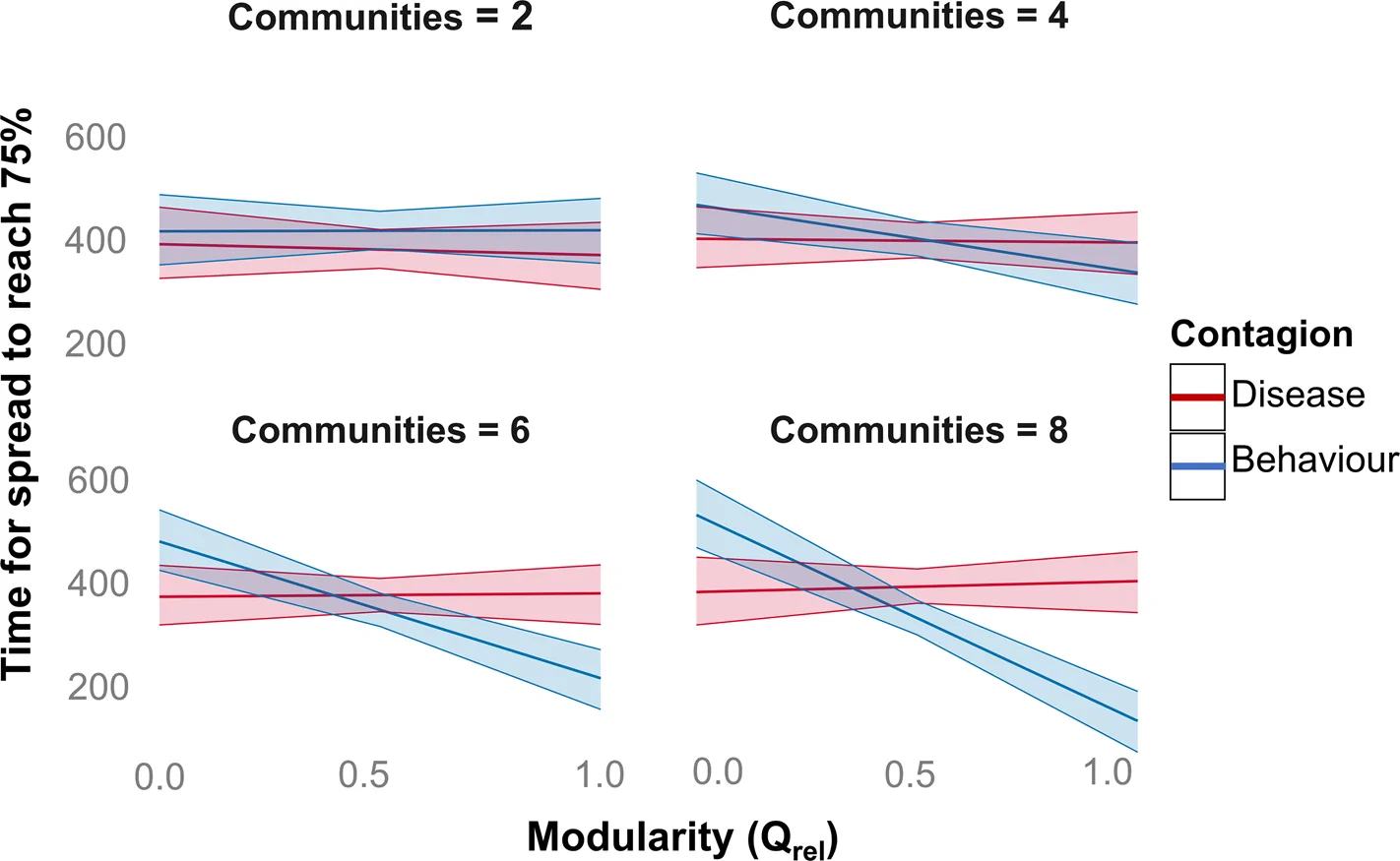
Influence of modularity (uncentred) on time to reach 75% spread across networks with varying numbers of communities (2, 4, 6 and 8) in disease contagions (red) and behavioural contagions (blue)—the shaded areas depict 95% confidence intervals.

Examination of the conditional main effects revealed further differences between disease and behavioural contagions. For disease contagions, for an average modularity, networks with more communities generally increased the time taken for the spread to reach 75% of the network (β=4.89,(95%CI:3.07,6.71); figure 5C), while at the average level of network fragmentation, modularity was negatively associated with spread time ( β=−85.55,(95%CI:−108.88,−61.56); figure 5A). As expected, larger network size was associated with increased spread time ( β=0.48,(95%CI:0.43,0.53); figure 5D), whereas higher network density was associated with decreased spread time, but credible intervals overlapped zero ( β=−4.80,(95%CI:−19.66,10.27); figure 5B). Full model results are in the supplementary material.

**Figure 5.**
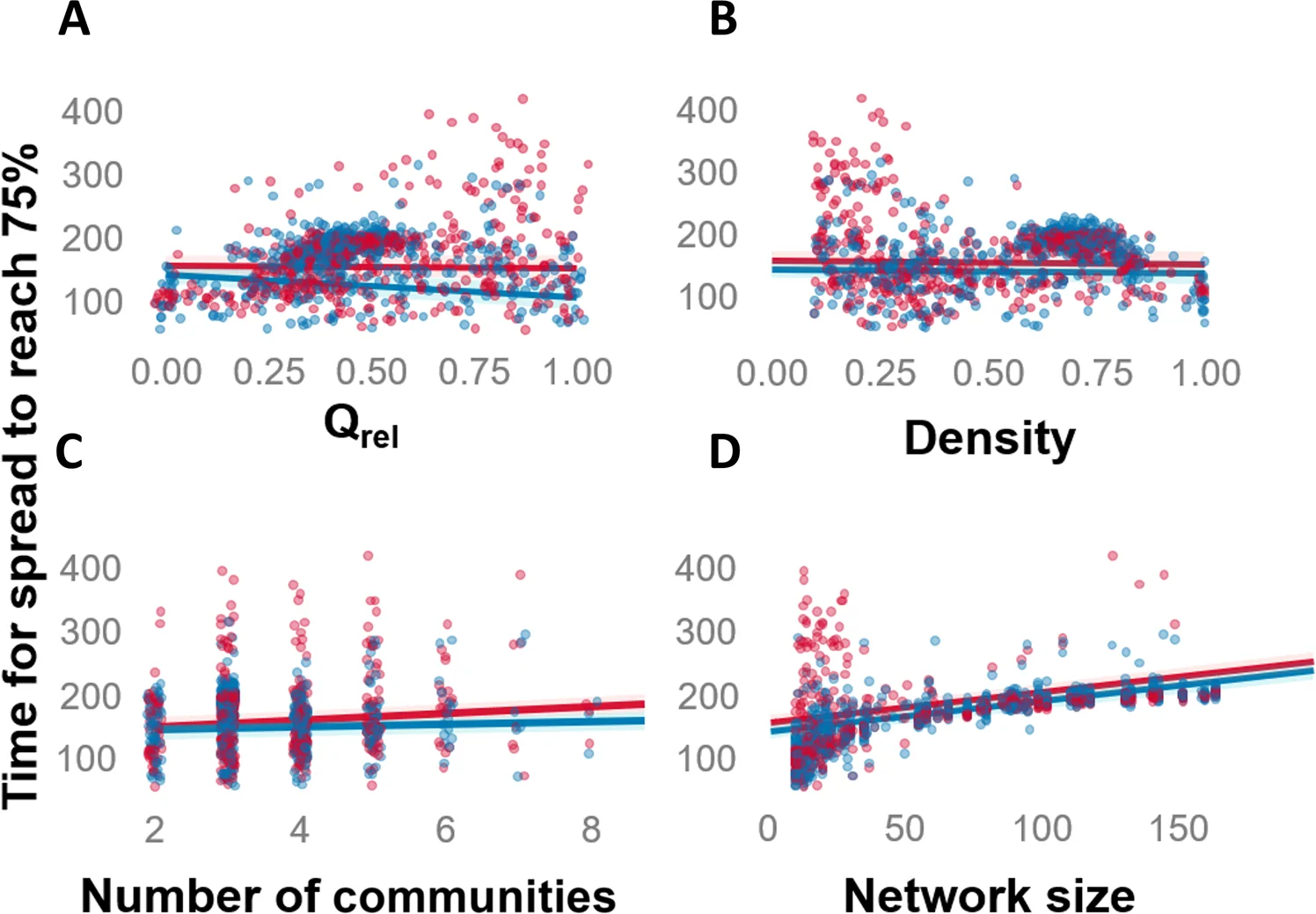
Partial effect plots illustrating how (A) modularity (Q_rel_), (B) density, (C) number of communities and (D) network size influence the time for contagion spread to reach 75% of the network in disease (red) versus behavioural (blue) contagions. Each point corresponds to the mean time for spread to reach 75% over 50 repeats in each network, and the lines represent the model's fitted partial effects and the shaded areas depict 95% confidence intervals. Modularity, network size and density were uncentred to their raw values for plotting. Since our regression model includes interaction effects, in (A), the number of communities is held at its mean and in (C), modularity is held at its mean.

Our additional model incorporating variation in community size revealed a three-way interaction with modularity ( β=27.02,(95%CI:7.99,45.51)), indicating that the effects of modularity are contingent on the distribution of community sizes, with stronger effects observed when communities are more similar in size. Our main finding remained consistent: behavioural contagions were less impeded by modular and fragmented network structures, as reflected by the negative three-way interaction between contagion type, modularity and number of communities ( β=−16.81,(95%CI:−19.73,−13.89)).

The overall (conditional) Bayesian *R*${}^{2}$ of our main model, which includes both fixed and random effects, was estimated at 0.273 (95% CI: 0.269, 0.279). The marginal Bayesian *R*${}^{2}$—reflecting only the contribution of fixed effects—was substantially lower at 0.142 (95% CI: 0.123, 0.168), suggesting that: (i) in our dataset of real-world networks, network fragmentation has a relatively modest effect on contagion outcomes, (ii) a substantial portion of the remaining variance is accounted for by the random effects in the model (i.e. variation among species and networks). To further decompose the variance attributable to random effects, we calculated intra-class correlation coefficients after accounting for fixed effects. These Intraclass Correlation Coefficients (ICCs) reflect a different variance decomposition from Bayesian *R^2^*, partitioning the remaining variance among species, network and residual components. Conditional on the fixed effects, species-level differences explained 64.3% of the remaining variance in spread time (95% CI: 55.3–73.0%), while network-level differences accounted for 4.3% (95% CI: 3.1–5.6%). The remaining 31.4% (95% CI: 23.8–39.3%) was residual variance, reflecting unexplained variation at the observation level beyond species and network structure. Figure S2 in the supplementary material illustrates how species differ in their mean contagion spread times.

## 4. Discussion

Our results reveal that as networks become increasingly fragmented and modular, contagion spread is delayed for simple contagions (e.g. infectious disease), whereas complex conformist contagions (e.g. information use and behaviour) can exploit dense community structure to spread to the majority of the network in less time.

Our results extend the theoretical findings of Evans *et al.* [32], which showed that highly subdivided social network architectures preferentially facilitate beneficial behavioural contagions over harmful pathogens, by testing these predictions across a large comparative dataset of empirical animal social networks and showing that the same structural patterns shape contagion dynamics in real systems, albeit with low explanatory power in empirical systems. The effect is most distinct in networks with the highest levels of community structuring—those that are both modular and fragmented. Using networks drawn from the ASNR [20], we find that as modularity increased, the time for 75% of individuals to become infected rose for disease contagions, but only in social networks with many, small communities and specifically when communities are similar in size. By contrast, behavioural contagions were not delayed and spread considerably faster than disease contagions when networks were fragmented and modular. However, while fragmentation and modularity in these empirical networks explains some of the differences in how these contagions play out, our study uniquely exemplifies that it accounts for only a small portion of the overall variation in outcomes, indicating that additional factors are important in shaping the dynamics of spread. Our findings demonstrate the value of using a taxonomically and behaviourally diverse sample of real-world network structures in our study as it reveals that the effects predicted by theoretical models are more variable and context-dependent in natural systems. While previous research has relied on theoretical network structures [32,47,56], our results inherently incorporate the complexity and heterogeneity present in natural social systems to demonstrate that these theoretical patterns play a limited but potentially important role in nature. Whereas Evans *et al.* [32] manipulated group size and modularity in synthetic networks of fixed size (200 nodes) to demonstrate these effects in theory, our study shows that the interaction between modularity and fragmentation continues to influence contagion dynamics across diverse empirical animal social networks spanning substantial variation in species identity, network size and community structure.

Our data-driven modelling approach demonstrates that the slowing effect of community structure on infectious disease spread is a realistic phenomenon in natural animal societies. Our findings complement epidemiological studies showing that modular structures facilitate rapid transmission within strongly connected groups while reducing between-group spread, effectively acting as a ‘brake’ on overall transmission [44,46,57,58]. By considering the interaction between network modularity and fragmentation, we extend the insights provided by [46] to show that group or subgroup size acts alongside the strength of subdivision between social units in determining epidemiological outcomes. We have focused on the overall (linear) effects of modularity and fragmentation in our analysis, but given that previous studies have provided evidence for nonlinear and threshold effects for the effect of modularity on contagion outcomes [46,59], this would be worthy of further study.

We show that the interaction between network fragmentation and contagion dynamics may have ecological and evolutionary implications, revealing how social structures can mediate trade-offs between social behaviour and disease spread. Although our simulations confirm that community structure and network fragmentation do interact to determine contagion outcomes, the: (i) pronounced effects being found only with high modularity and/or fragmentation, and (ii) modest effect sizes and relatively low marginal Bayesian $R^{2}$ ( fixed network structural predictors) compared with the higher overall (conditional) $R^{2}$ that incorporates species and network-level variability indicate that other factors drive much of the variation in contagion spread in real-world networks. A substantial part of this unexplained variation may arise from smaller networks, where stochasticisity and sparse connections amplify the unpredictability of contagion outcomes. Additionally, stochastic variation in initial seeding may further contribute to the observed variability, as different starting nodes can lead to markedly different transmission trajectories even within the same network. Future work should consider how variation in node-level centrality and seeding position interact to shape the distinct transmission dynamics of disease versus social contagions [60].

Here, we spread social behaviour with a conformist learning rule, which has been documented across several species [36,37,61]. In our study, the behavioural contagion traverses modular boundaries more efficiently, even when the same network structure impedes the spread of a simple contagion. This may be owing to social reinforcement, where tight-knit subgroups with high clustering repeatedly expose individuals to the behaviour, making it easier for a complex contagion which requires multiple exposures for transmission, to spread [56,62]. Such dynamics allow complex contagions to leverage community structure to enhance spread through social reinforcement of multiple clustered contacts, described by Centola [63] as a ‘snowball’ strategy. Consistent with this, complex health behaviours have been shown to spread more effectively on clustered than random networks in human social systems [64]. Network measures developed for complex contagions, such as complex path length and complex centrality, capture how reinforcement pathways facilitate spread across communities [65]. Exploring these features in empirical animal social networks could help reveal how the evolutionary ecology of animal social systems shapes complex contagion dynamics. Animal social systems exhibit great diversity, with social networks varying significantly in their community structure [18,66,67]. Notably, whereas Evans *et al.* [32] established these mechanisms in theoretical networks with manipulated group sizes and modularity, our results demonstrate that these same structural properties occur in empirical animal social systems and may similarly mediate the balance between behavioural and disease contagions, albeit less consistently.

Our results indicate that modular networks only balance the trade-off between behavioural and disease contagions when highly fragmented into many communities. This distinction is important because: (i) different social systems can vary considerably in the size of their groups or subgroups (e.g. squamate reptiles can have as few as two to as many as 20 000 conspecifics in a group [68]), (ii) modularity can arise at different scales—either through discrete, weakly connected groups at the population level or through hierarchical structuring within groups, as seen in multi-level societies [44,69,70]. In such systems, subgroup formation may help balance evolutionary pressures living in these stable social groups, and nested substructures within larger groups may influence contagion dynamics at higher social scales.

While our findings are encouraging, several modelling limitations warrant consideration. We spread social behaviour as a complex contagion, using conformist learning, yet transmission dynamics can depend on the mode of information and behaviour transmission. Beck *et al.* [71] showed that adoption driven by the sum and strength of informed contacts favours early uptake by highly connected individuals, whereas ratio-based transmission does not. Hence, alternative social-behaviour mechanisms could yield outcomes different to our conformist model. If behaviour spread followed simpler rules, such as proximity-triggered activity [71], network effects might resemble those of simple contagions [25]. Similarly, the spread of infection could resemble a complex contagion, especially if following a nonlinear dose–response relationship [72]. We calibrate transmission for simple and complex contagions to be equivalent on random networks in order to isolate the effect of network structure. However, behavioural and pathogen transmission may operate on different absolute time scales in nature [35]. Here, we focus on whether their dynamics are comparable, particularly in relation to modularity and fragmentation, but future work could incorporate different transmission time scales to test whether these structural effects remain consistent when absolute rates of spread differ. Separately, it would be interesting to examine how behavioural and pathogen contagions interact or compete when they co-occur, as feedbacks between behaviour and disease dynamics are known to shape transmission outcomes [73]. Additionally, because spread times were analysed conditional on reaching the 75% adoption threshold, variation in the proportion of simulations successfully reaching this threshold across networks may further contribute to the observed variability in spread times. Finally, our SI model provides valuable insights into the early stages of contagion uptake, but future extensions incorporating recovery (SIR) or reintroducing infected individuals into the susceptible pool (SIS) could shed light on how social structure differentially influences the long-term dynamics of simple versus complex contagions [74].

Our sample of networks from the ASNR, although normalized across systems, includes diverse network types and edge definitions (e.g. spatial proximity to direct physical contact). While allowing us to generalize across animal social network types, this does introduce variability that likely added uncertainty to our conclusions [19,75]. Namely, a limitation of our study is the inclusion of small (<20 nodes) and sparse (low-density) networks, which produced greater variance in spread times and inflated Q_rel_ values owing to instability in community detection [76,77]. Although we explored this in the supplementary material and found that models restricted to larger or denser networks were qualitatively similar in the direction of effects, higher variance in small or sparse networks reduces precision. Because many real-world animal social networks are inherently small—often owing to sampling constraints—this limitation is difficult to avoid entirely, but it should be considered when interpreting results. Also, it should be noted that, because simulations were conducted on the largest connected component of each network, isolates and small components (representing socially or spatially disconnected individuals) were excluded. This may underestimate overall modularity and fragmentation relative to the full network, and our analyses therefore focus on within-component modular structure. Expanding the range of networks in the ASNR will enable more representative comparative analyses across animal social systems.

### 4.1. Conclusions

Our study demonstrates that highly fragmented and modular networks can differentially influence contagion spread, with complex contagions propagating across subgroup boundaries more effectively than simple contagions extending the theoretical predictions of [32] to empirical social systems. This differential effect has important evolutionary implications, suggesting that modular structures with many subgroups may enhance fitness of individuals that form these types of social networks by improving access to beneficial social behaviours while mitigating the risk of acquiring infection. However, we do identify a relatively small effect size showing that these network features have a weaker effect in real-world systems than theoretical models like in [32], revealing the importance of data-driven modelling in translating theory into practical applications in social ecology and evolution. Our findings have practical implications: for example, management interventions aimed at fragmenting social networks, such as targeted hunting, fencing or habitat manipulation [78], may slow disease spread but will also disrupt behavioural contagion processes, necessitating a careful assessment of unintended (behavioural) effects of these interventions. Future work could track individual-level behaviours and extend the framework to interacting or competing contagions, helping clarify how individual roles and network structure jointly shape transmission and its ecological and evolutionary consequences.

## Supplementary Material



## Data Availability

All code required to reproduce the analysis, social networks used and dataset generated from simulations are available at Zenodo Repository [51]. Supplementary material is available online [79].
