# Supplementary material for: Fragmented social networks promote complex behavioural contagions over infectious disease spread
Source: Proc Biol Sci. 2026 Oct 28;293(2081):20261424. doi: 10.1098/rspb.2026.1424 (PMC13725535; doi:10.1098/rspb.2026.1424)
Supplement: Supplementary file 1 [file rspb.2026.1424.zip › rspb.2026.1424/rspb20261424_si_001.pdf]

Electronic Supplementary Material

Fragmented social networks promote complex behavioural contagions over infectious disease spread

Nitara Wijayatilake, Shweta Bansal, Amy B. Pedersen, Matthew Silk

Proceedings of the Royal Society B
DOI: 10.1098/rspb.2026.1424

- Modularity Calculations
- Robustness model(s) results
- Collinearity diagnosis
- Random Effects
- Conformist Adoption function
- Model check plot

Modularity Calculations

Modularity (Q) was calculated following Newman's (2004) definition [1]:

$$Q = \frac{1}{2m} \sum_{i,j} \left(A_{ij} - \gamma \frac{k_i k_j}{2m} \right) \delta(c_i, c_j) \tag{1}$$

where m is the number of edges, A_{ij} is the weight of the A adjacency matrix in row i and column j, k_i is the degree of node i, k_j is the degree of node j, c_i is the component of node i, c_j is the component of node j, the sum goes over all pairs of nodes i and j, and $\delta(x, y)$ equals 1 if $x = y$ and 0 otherwise.

Because the maximum modularity (Q_{max}) depends on the size and number of links in the networks, direct comparisons of raw modularity values across networks can be misleading. We therefore computed relative modularity (Q_{rel}), by normalising observed modularity (Q) by its theoretical maximum (Q_{max}), calculated using [2]:

$$Q_{\text{max}} = \sum_{k=1}^{K} \left(\frac{L_k}{L} \left(1 - \frac{L_k}{L} \right) \right) \tag{2}$$

where L_k is the number of edges within subgroup k, L is the total number of edges in the network, and K is the number of subgroups. Relative modularity was then defined as:

$$Q_{\text{rel}} = \frac{Q}{Q_{\text{max}}} \tag{3}$$

Other statistical models

To assess the robustness of our main findings, we evaluated alternative statistical models. These models allow us to confirm that the observed differences between behavioural and disease contagion dynamics are not driven by a single modelling choice.

- Heterogeneous variance model
- Standard hierarchical model

- High size–density threshold model
- Model with no *Camponotus fellah*
- Subgroup cohesion instead of Q_{rel}
- Module Size Variation model
- 2 seeds model
- 3 seeds model
- $R_0 = 3$ model
- Time to 50% infection model (different endpoint)

Results from all the above models are shown in Table 1 and 2. Notably, the three-way interaction among contagion type, modularity, and number of communities remained negative and statistically clear in all models we ran, confirming that social contagions spread more effectively in fragmented and modular networks than disease contagions do (Supplementary Figure 4).

Standard hierarchical model

We ran two main models: a heterogeneous variance model (presented in the main text) and a standard hierarchical model. The standard hierarchical model incorporated nested random effects for species within networks, but assumed a constant residual variance across all groups while the heterogenous variance model allowed residual standard deviation to vary across species and networks. Model fit was assessed using Leave-One-Out Cross-Validation (LOO). For each model, we computed the Expected Log Predictive Density (ELPD) and the LOO Information Criterion (LOOIC), where a higher ELPD and a lower LOOIC indicate better predictive performance [3]. Model comparisons were based on the difference in ELPD along with its associated standard error. The model with higher ELPD (and correspondingly lower LOOIC) was selected. The heterogeneous variance model showed better predictive performance than the standard hierarchical model, with higher ELPD (−305,198.1, SE = 269.2) and lower LOOIC (610,396.2, SE = 538.4), compared with the standard hierarchical model (ELPD = −311,196.1, SE = 347.1; LOOIC = 622,392.1, SE = 694.2). The heterogeneous variance model also used more effective parameters ($p_{\mathrm{LOO}} = 689.7$) than the standard hierarchical model ($p_{\mathrm{LOO}} = 561.5$). The key three-way interaction remained consistent across both model structures, supporting the robustness of the main result.

Model with no *Camponotus fellah*

To test the robustness of our findings, we re-estimated the Bayesian regression model excluding ant (*Camponotus fellah*) networks, which accounted for a disproportionately large number of cases ($n = 221$) due to repeated sampling in the original study. Results were consistent with the full model (Table 2). The direction and magnitude of the three-way interaction between modularity, number of communities and contagion type ($\beta = -4.49$, 95% CI: −6.28, −2.66) supports the conclusion that behavioural contagions consistently reach 75% of the network faster than disease contagions at high levels of both modularity and fragmentation.

Subgroup cohesion model

We also ran a version of the model replacing Q_{rel} with subgroup cohesion, defined as the proportion of contacts occurring within subgroups. In contrast to the modularity models, we found no clear evidence that subgroup cohesion differentially affected behavioural and disease contagions, with interaction effects overlapping zero ($\beta = 1.04$, 95% CI: −1.52, 4.29).

High size–density threshold model

We ran an additional model restricting analyses to networks with at least 20 nodes and density ≥ 0.15 to assess the influence of small or sparse networks on model results. The model specification was identical to

the heterogeneous variance model presented in the main text, but applied to the reduced dataset ($n = 360$ networks). Although the effect weakened, results were qualitatively consistent with the main model in both the magnitude and direction of effects, including a negative and statistically clear three-way interaction between contagion type, modularity, and number of communities ($\beta = -2.70$, 95% CI: $[-6.24, -0.85]$). This suggests that the main conclusions are robust to the exclusion of small or sparse networks.

Module size variation model

We extended the model by including variation in community size (measured as the coefficient of variation in module size) and its three-way interaction with relative modularity (Qrel) and contagion type. The main result remained unchanged: behavioural contagions traversed highly modular and fragmented networks more effectively than disease contagions ($\beta = -17.58$, 95% CI: $[-20.90, -14.26]$). However, this effect depended on variation in module size. The positive three-way interaction between module size variation, Qrel, and contagion type ($\beta = 27.02$, 95% CI: $[7.99, 45.51]$) indicates that the advantage of behavioural contagions is strongest when modules are more similar in size, and weakens as variation in module size increases.

Endpoint = 50% infection

We conducted an additional sensitivity analysis using time to 50% network coverage as the response variable, rather than the primary threshold of 75%. Results were qualitatively consistent: behavioural contagions traversed highly modular and fragmented networks more effectively than disease contagions even to reach only half of individuals in the network rather than the vast majority (75%) ($\beta = -16.56$, 95% CI: $[-19.19, -13.90]$).

2-seed model

We repeated the simulations using the same method as in the main text, but seeding the contagion(s) with 2 starting nodes. Results were similar: behavioural contagions more effectively traversed high modularity and a higher number of communities than disease contagions ($\beta = -25.26$, 95% CI: -29.43, -21.03).

3-seed model

We repeated the simulations using the same method as in the main text, but seeding the contagion(s) with 3 starting nodes. Results were similar: behavioural contagions more effectively traversed high modularity and a higher number of communities than disease contagions ($\beta = -9.33$, 95% CI: -11.32, -7.36).

$R_0 = 3$ model

We repeated the simulations using the same method as in the main text, but with transmission probability calibrated to $R_0 = 3$ rather than $R_0 = 1.5$. Results were qualitatively consistent: behavioural contagions traversed highly modular and fragmented networks more effectively than disease contagions ($\beta = -7.38$, 95% CI: $[-9.50, -5.26]$).

**Predictors**	**Heterogeneous variance**	**Standard hierarchical**	**High size–density model**
Intercept	136.37 (121.31, 151.40)	146.96 (134.10, 160.94)	160.96 (141.89, 179.44)
Sigma Intercept	4.03 (3.88, 4.18)	63.34 (62.95, 63.72)	3.98 (3.89, 4.06)
type [social contagion]	-7.10 (-9.43, -4.72)	-21.33 (-23.86, -18.84)	6.66 (4.14, 9.23)
Q_{rel} (centred)	-85.55 (-108.88, -61.56)	-50.49 (-71.01, -31.43)	-5.47 (-45.70, 35.59)
Number of communities	4.89 (3.07, 6.71)	3.43 (1.88, 5.05)	0.18 (-1.80, 2.13)
Density (centred)	-4.80 (-19.66, 10.27)	-5.00 (-17.08, 7.30)	-42.80 (-60.20, -25.81)
Network size (centred)	0.48 (0.43, 0.53)	0.46 (0.42, 0.50)	0.38 (0.34, 0.42)
type × Q_{rel}	17.84 (7.66, 27.80)	-11.18 (-19.99, -1.86)	-2.69 (-18.37, 13.31)
type × # communities	-2.31 (-2.98, -1.65)	-1.18 (-1.88, -0.49)	-2.99 (-3.75, -2.25)
Q_{rel} × # communities	27.32 (21.42, 33.07)	20.17 (15.31, 25.35)	2.64 (-6.54, -11.60)
type × Q_{rel} × # communities	-16.87 (-19.79, -13.99)	-12.30 (-14.79, -9.81)	-2.70 (-6.24, -0.85)
Bayesian R^2 (conditional)	0.273 (0.269, 0.279)	–	–
Bayesian R^2 (marginal)	0.142 (0.123, 0.168)	0.338 (0.335, 0.341)	0.319 (0.310, 0.327)
Random effects (SD)			
Species	55.18 (44.94, 67.51)	45.56 (37.10, 55.74)	51.86 (40.11, 67.14)
Network	14.16 (13.15, 15.23)	12.27 (11.44, 13.13)	9.89 (8.88, 11.01)
Species (on σ)	0.61 (0.50, 0.73)	–	0.24 (0.18, 0.32)

**Predictors**	**No *C. fellah***	**Subgroup cohesion**	**Module size variation**
Intercept	105.07 (80.74, 129.47)	71.80 (2.05, 117.26)	137.80 (121.48, 154.39)
Sigma Intercept	3.72 (3.52, 3.92)	2.79 (-0.85, 4.09)	4.03 (3.86, 4.18)
type [social contagion]	-21.61 (-23.82, -19.35)	12.33 (1.73, 19.50)	-5.86 (-8.28, -3.42)
Q_{rel} (centred)	-128.18 (-190.63, -66.81)	–	-85.03 (-110.11, -59.45)
Subgroup cohesion	–	34.79 (-1.84, 81.62)	–
Module size variation	–	–	-3.02 (-10.32, 4.46)
Number of communities	10.30 (4.95, 15.68)	7.95 (-1.02, 15.26)	4.81 (2.85, 6.73)
Density (centred)	24.90 (-15.45, 64.36)	-7.57 (-30.86, 9.74)	-5.88 (-19.53, 8.19)
Network size (centred)	0.37 (0.24, 0.50)	0.55 (0.30, 1.10)	0.48 (0.43, 0.53)
type × Q_{rel}	19.30 (12.88, 25.71)	–	19.09 (7.53, 30.50)
type × subgroup cohesion	–	-23.42 (-38.39, -0.16)	–
type × module size variation	–	–	13.64 (10.07, 17.30)
type × # communities	0.25 (-0.30, 0.81)	-3.62 (-5.95, 0.93)	-2.81 (-3.51, -2.12)
Q_{rel} × # communities	23.55 (8.82, 38.37)	–	27.40 (21.18, 33.88)
Subgroup cohesion × # communities	–	-0.01 (-12.87, 13.16)	–
type × Q_{rel} × # communities	-4.49 (-6.28, -2.66)	–	-17.58 (-20.90, -14.26)
type × subgroup cohesion × # communities	–	1.04 (-1.52, 4.29)	–
Bayesian R^2 (marg.)	0.487 (0.481, 0.493)		0.275 (0.270, 0.279)
Random effects (SD)			
Species	48.34 (36.73, 62.78)	32.37 (3.22, 53.31)	54.81 (44.57, 67.33)
Network	41.73 (38.26, 45.54)	21.57 (1.33, 30.12)	14.10 (13.11, 15.17)
Species (on σ)	0.54 (0.39, 0.73)	0.26 (0.13, 0.42)	0.60 (0.50, 0.72)

**Predictors**	**2 seeds**	**3 seeds**	$R_0 = 3$	**50% threshold**
Intercept	274.00 (245.95, 301.34)	120.06 (104.85, 135.04)	113.45 (95.15, 131.09)	94.92 (81.06, 108.66)
Sigma Intercept	110.92 (110.23, 111.64)	51.70 (51.36, 52.03)	3.82 (3.65, 4.00)	3.97 (3.85, 4.09)
type [social contagion]	-34.21 (-38.62, -29.63)	-8.83 (-10.99, -6.69)	-1.98 (-4.02, 0.04)	22.72 (20.51, 24.94)
Q_{rel} (centred)	-211.54 (-261.17, -159.94)	-109.90 (-136.27, -84.02)	-117.53 (-148.40, -86.55)	-49.38 (-67.12, -30.58)
Number of communities	10.20 (6.15, 14.22)	5.59 (3.39, 7.85)	6.88 (4.54, 9.31)	3.80 (2.39, 5.23)
Density (centred)	-64.73 (-96.29, -33.62)	1.81 (-15.16, 18.98)	6.90 (-12.31, 25.53)	6.52 (-4.12, 17.84)
Network size (centred)	0.87 (0.77, 0.99)	0.47 (0.42, 0.53)	0.49 (0.43, 0.56)	0.46 (0.42, 0.50)
type × Q_{rel}	22.16 (7.06, 36.94)	22.33 (15.20, 29.48)	28.34 (20.37, 36.27)	2.22 (-7.38, 11.71)
type × # communities	-2.37 (-3.63, -1.16)	-1.20 (-1.79, -0.62)	-1.74 (-2.31, -1.17)	-1.77 (-2.38, -1.16)
Q_{rel} × # communities	56.15 (44.14, 68.17)	26.99 (20.62, 33.24)	27.29 (19.78, 34.61)	20.94 (16.36, 25.37)
type × Q_{rel} × # communities	-25.26 (-29.43, -21.03)	-9.33 (-11.32, -7.36)	-7.38 (-9.50, -5.26)	-16.56 (-19.19, -13.90)
Bayesian R^2	0.375 (0.371, 0.378)	0.468 (0.465, 0.472)	0.464 (0.459, 0.468)	0.311 (0.306, 0.315)
				Random effects (SD)
Species	89.28 (71.24, 111.47)	49.56 (39.87, 61.34)	61.81 (50.64, 75.70)	49.07 (40.32, 60.46)
Network	31.93 (29.93, 34.15)	17.45 (16.34, 18.62)	19.45 (18.28, 20.75)	10.63 (9.89, 11.39)
Species (on σ)	–	–	0.71 (0.59, 0.85)	0.45 (0.37, 0.55)

Collinearity diagnosis

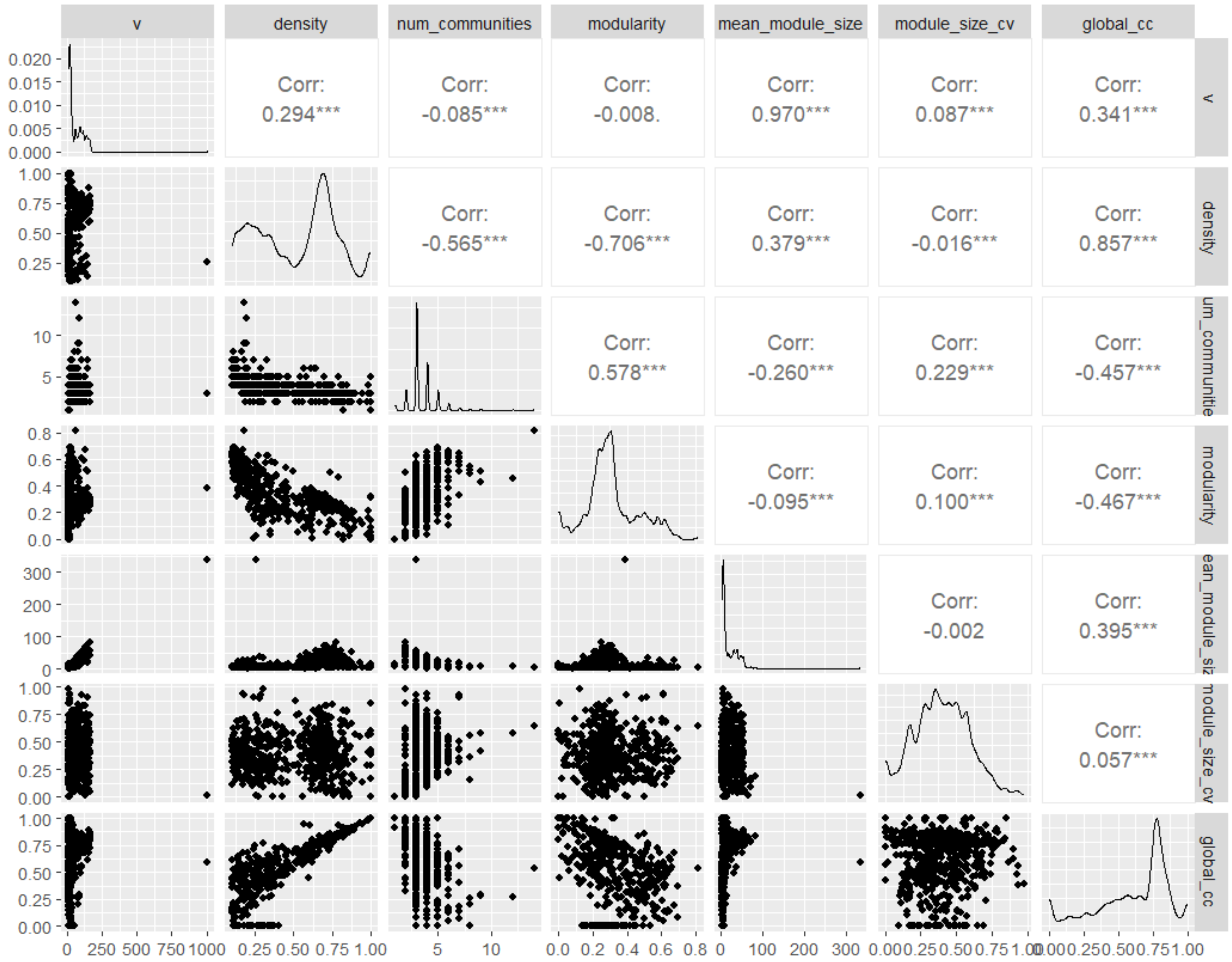

Supplementary Figure 1. Pairwise relationships among network size (v), density, number of communities, modularity, mean module size, module size variation (coefficient of variation of module sizes), and global clustering coefficient (global CC). Pearson correlations are shown in the upper panels, scatterplots in the lower panels, and distributions on the diagonal. Variables with correlations > 0.8 (global CC and mean module size) were excluded from downstream analyses.

Species-level random effects

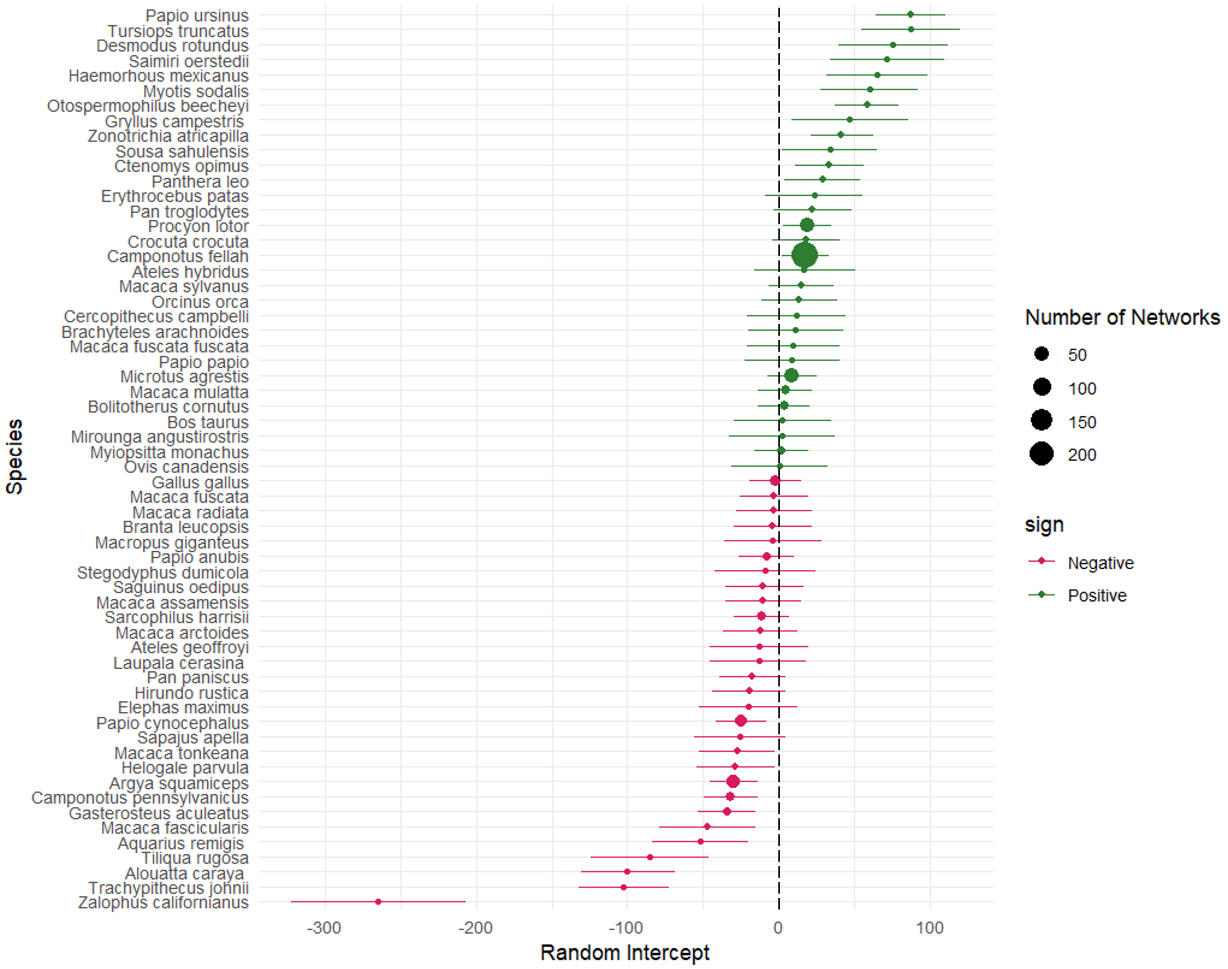

Supplementary Figure 2. Species-level random intercepts from the Bayesian model, illustrating variation among species in the baseline time for contagion spread. Circles show each species' posterior mean intercept, and horizontal lines represent 95% credible intervals. Circle size is proportional to the number of networks contributed by that species, and circle colour indicates whether the random intercept is positive (green) or negative (pink) relative to zero.

Conformist Adoption function

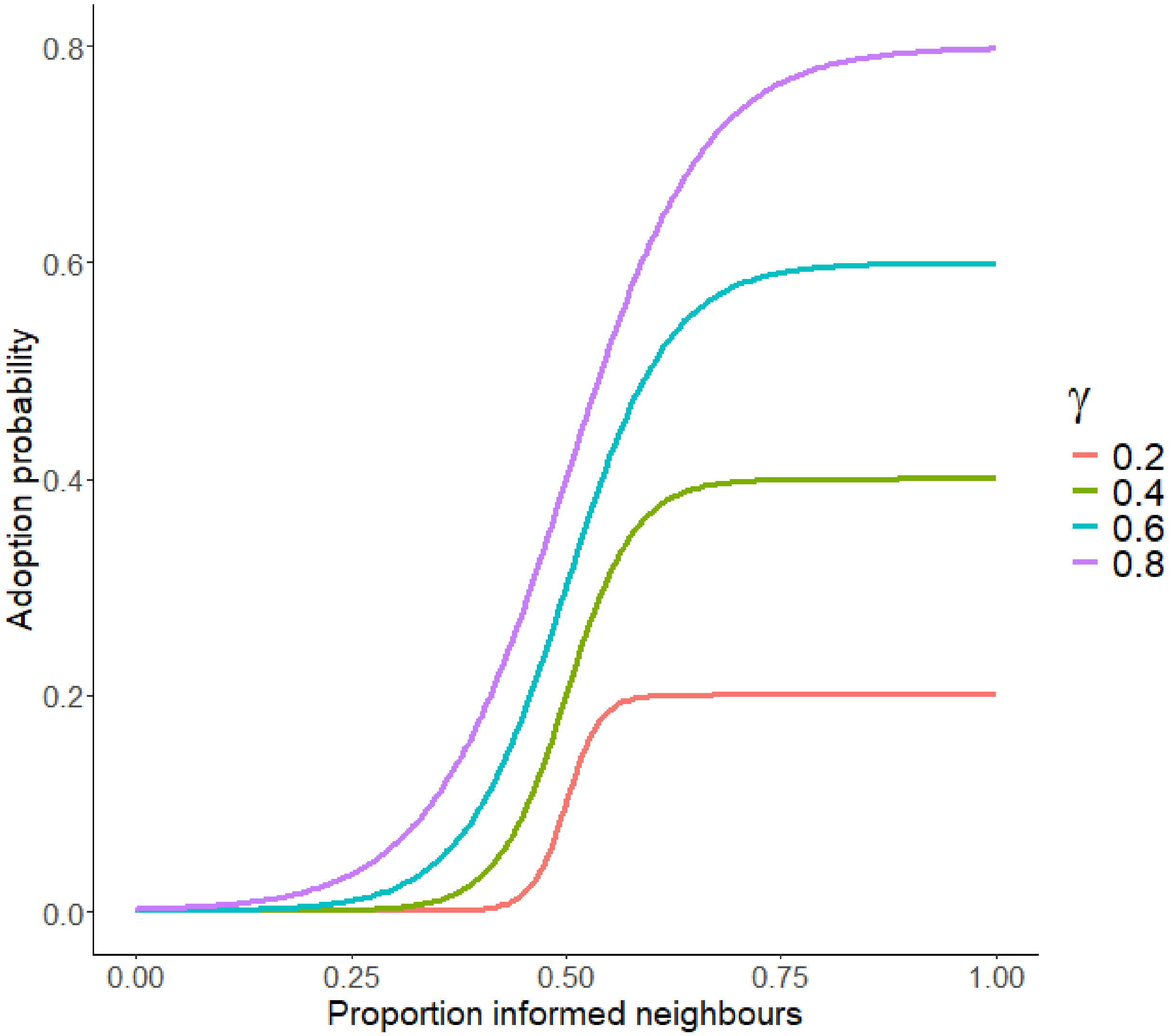

Supplementary Figure 3. Conformist adoption function illustrating the relationship between the proportion of informed neighbours and adoption probability for varying values of the scaling parameter γ. While γ affects the overall magnitude of adoption probabilities, the sigmoidal form of the function, determined by η (0.001) and ψ (10), remains intact, indicating that the process retains its non-linear, reinforcement-driven dynamic. The low value of η allows only minimal asocial learning, so adoption remains primarily driven by social reinforcement.

Posterior predictive model check

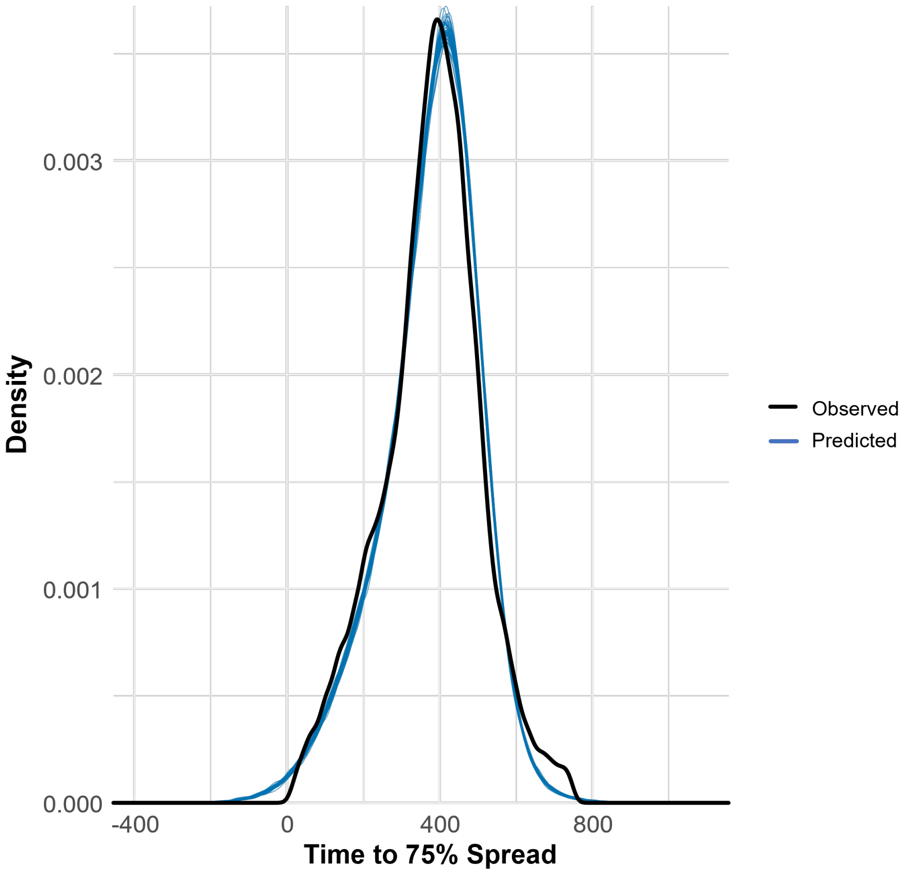

Supplementary Figure 4. Posterior predictive check using a density overlay: the black curve shows the observed distribution of time to 75% spread, and the blue curves represent 30 posterior predictive draws from the Bayesian model, demonstrating model fit.

References

1. Clauset, A.; Newman, M. E. J.; Moore, C. Finding community structure in very large networks. *Physical Review E*, 2004, 70, 066111.

2. Sah, P.; Leu, S. T.; Cross, P. C.; Hudson, P. J.; Bansal, S. Unraveling the disease consequences and mechanisms of modular structure in animal social networks. *Proceedings of the National Academy of Sciences*, 2017, 114(16), 4165–4170.

3. Vehtari, A.; Gelman, A.; Gabry, J. Practical Bayesian model evaluation using leave-one-out cross-validation and WAIC. *Statistics and Computing*, 2017, 27(5), 1413–1432.
